# Learning contextual gene set interaction networks of cancer with condition specificity

**DOI:** 10.1186/1471-2164-14-110

**Published:** 2013-02-19

**Authors:** Sungwon Jung, Michael Verdicchio, Jeff Kiefer, Daniel Von Hoff, Michael Berens, Michael Bittner, Seungchan Kim

**Affiliations:** 1 Integrated Cancer Genomics Division, Translational Genomics Research Institute, Phoenix, Arizona, USA; 2 School of Computing, Informatics and Decision Systems Engineering, Arizona State University, Tempe, USA; 3 Pharmaceutical Genomics Division, Translational Genomics Research Institute, Phoenix, Arizona, USA; 4 Clinical Translational Research Division, Translational Genomics Research Institute, Phoenix, Arizona, USA; 5 Cancer and Cell Biology Division, Translational Genomics Research Institute, Phoenix, Arizona, USA; 6 Computational Biology Division, Translational Genomics Research Institute, Phoenix, Arizona, USA

**Keywords:** Molecular context, Cancer, Condition-specificity, Gene regulatory network, Glioblastoma

## Abstract

**Background:**

Identifying similarities and differences in the molecular constitutions of various types of cancer is one of the key challenges in cancer research. The appearances of a cancer depend on complex molecular interactions, including gene regulatory networks and gene-environment interactions. This complexity makes it challenging to decipher the molecular origin of the cancer. In recent years, many studies reported methods to uncover heterogeneous depictions of complex cancers, which are often categorized into different subtypes. The challenge is to identify diverse molecular contexts within a cancer, to relate them to different subtypes, and to learn underlying molecular interactions specific to molecular contexts so that we can recommend context-specific treatment to patients.

**Results:**

In this study, we describe a novel method to discern molecular interactions specific to certain molecular contexts. Unlike conventional approaches to build modular networks of individual genes, our focus is to identify cancer-generic and subtype-specific interactions between contextual gene sets, of which each gene set share coherent transcriptional patterns across a subset of samples, termed *contextual gene set*. We then apply a novel formulation for quantitating the effect of the samples from each subtype on the calculated strength of interactions observed. Two cancer data sets were analyzed to support the validity of condition-specificity of identified interactions. When compared to an existing approach, the proposed method was much more sensitive in identifying condition-specific interactions even in heterogeneous data set. The results also revealed that network components specific to different types of cancer are related to different biological functions than cancer-generic network components. We found not only the results that are consistent with previous studies, but also new hypotheses on the biological mechanisms specific to certain cancer types that warrant further investigations.

**Conclusions:**

The analysis on the contextual gene sets and characterization of networks of interaction composed of these sets discovered distinct functional differences underlying various types of cancer. The results show that our method successfully reveals many subtype-specific regions in the identified maps of biological contexts, which well represent biological functions that can be connected to specific subtypes.

## Background

Many computational and mathematical techniques have been developed to infer molecular patterns of biological and translational interest from gene expression data profiled from human tumors. As most of these methodologies are highly dependent on simple correlation of changes in mRNA abundance as the primary measure of relatedness, they are intrinsically limited in their sensitivity and specificity by the highly heterogeneous, idiosyncratic nature of tumor gene expression patterns. Early expectations were that the molecular pathology of tumors arising from a particular tissue of origin would show striking similarities due to very common sets of oncogenic molecular processes accounting for each such tumor type’s initiation and progression. The finding that samples of tumors taken at different points in the course of an individual’s disease were more similar to each other than to any other tumor in the study was therefore quite surprising and stands as a noteworthy finding from early expression profiling studies [1]. One tumor type, chronic myelogenous leukemia (CML), has been found to have a large number of genes behaving in a very homogeneous fashion [2], however this kind of behavior has been the exception rather than the rule. The relative homogeneity of CML is probably in line with the expectation that a cancer type would exhibit high homogeneity if there was a high similarity in the process of oncogenesis. In the case of CML, it is true that the means of transformation is simple and constant. As the biochemical mechanisms of tumor growth and survival have been subjected to ever more detailed analysis, it has become clear that for most tumor types there is substantial variation in how tumors use available normal and altered cellular functions to achieve relentless growth and disproportionate survival.

In recent studies, hence, the identification of genomic patterns that are specific to certain biological contexts is gaining more interest as the heterogeneity in biological data becomes better embraced. *Biological contexts* of interest can be derived from subtypes of diseases or different clinical outcomes within the same subtype, such as responses to therapy. One of the early approaches to identify context-specific patterns involved searching for the co-regulated sets of genes and depicting the relationships between the gene sets and the biological or clinical characterization of samples. Gasch and Eisen [3] used a modified fuzzy *k*-means clustering method to find gene sets and showed correlation between those gene sets and the experimental conditions that determined how yeast cells respond to environmental changes. Segal et al. [4] used existing knowledge sources as well as clustering techniques to find gene sets that are either functionally co-related or coherently expressed in each set, then determined their specificity to particular types of tumors.

More recently, new strategies to identify context specificity of biological interactions are being proposed, as it is being widely accepted that biological interactions are coordinated in systematic ways but distinctively so, depending on biological contexts. These approaches are often based on network models representing biological interactions. Identifying context specificity in biological interactions can reveal environmental conditions under which the activities of components of biological networks vary, and this can make significant contribution to reinforcing confidence in the network as well as to improving the understanding of more exact mechanisms of transcriptional or translational regulations. Several studies have shown that it is possible to identify context-specific activity in known biological networks [5-8]. In these studies, biological networks were built from existing prior knowledge, and context-specific gene expression or protein expression data was used to annotate biological interactions’ context specificities. While this approach is useful for understanding the context specificity in already characterized networks, its utility is limited by the scope of the network as context-specificity is identified only for those interactions known from prior knowledge. Considering such limitation, a more desirable approach is to simultaneously identify biological interaction networks and their context specificity from high-throughput data. Grzegorczyk et al. [9] proposed a method to learn a non-homogeneous Bayesian network that can represent multiple different conditions, by using a mixture model that unifies networks of different conditions. Such approach is ideal in situations where enough amount of samples and computing resources are available. However, the scalability of its application is significantly limited in practice due to the complexity of the model. Indeed, their study only show the results from networks of up to 11 genes. Another possible approach is applying conventional network learning methods to subtype-specific samples and comparing the results. However, this approach requires many samples for each condition to achieve reliable results, and comparing inferred models from different conditions can be arguable especially when different amount of samples were available across conditions.

In this study, we propose a novel method to learn contextual gene set interaction networks that can represent maps of functional modules in target biological systems with statistical interactions between gene sets, and identify condition/subtype-specificity of inferred interactions. Our method comprises two novel approaches. The first is using context-specific gene sets as nodes of networks instead of individual genes, and the second is measuring condition-specificity with a formulation based on the probabilistic graphical model. Using gene sets instead of individual genes as nodes in networks can significantly increase the scalability of the application. To identify context-specific gene sets, we use a computational method that we have developed to model context-specific genomic regulations [10-12]. Identified gene sets, termed *contextual gene sets*, have coherent expression patterns specific for a subset of samples where they have statistically significant coherency. This property helps to identify networks of gene sets and relevant condition-specificity. Another novel aspect of our approach is using the conventional homogeneous Bayesian network model to learn networks and to measure condition-specificity. Using homogeneous network models requires significantly less computational cost than using non-homogeneous mixture models, compared to Grzegorczyk et al. [9], thus it is more scalable and can be applied to problems of larger scales. For example, we found limited application of the non-homogeneous mixture Bayesian network model proposed by Grzegorczyk et al. [9], while the conventional homogeneous Bayesian network model has been widely used for applications of varying sizes, from about one hundred [13] to almost one thousand genes [14,15]. However, such homogeneous model does not represent any condition specificity by itself. To overcome this limitation, we designed a novel formulation to quantitate the effect of the samples from different conditions/subtypes on the formation of networks to measure the degree and the statistical significance of condition specificity. The brief results of a simulation study is also given to show the feasibility of identifying condition-specificity.

Two cancer data sets were used as applications to show the benefit of identifying condition/subtype-specificity, 1) a refractory cancer gene expression data with 113 cancer patient samples of 32 different tissue types [16], and 2) The Cancer Genome Atlas (TCGA) glioblastoma multiforme (GBM) gene expression data. Each resultant contextual gene set interaction network shows both cancer-generic and subtype-specific interactions. The comparison to the result using a conventional biclustering-based approach, using a refractory cancer data set, we show the proposed method is much more sensitive in identifying context-specific interactions. We also found that the identified cancer-generic and subtype-specific sub-networks have different functional roles. Besides the comparison of functional annotations, we also related the identified subtype-specific interactions to supporting evidence from other knowledge sources. These results show that our approach to identify condition specificity in learned networks can provide novel information about biological functions specific to the given conditions.

## Results and discussion

### Overview of learning contextual gene set interaction networks and identifying condition specificity

**Figure 1:**
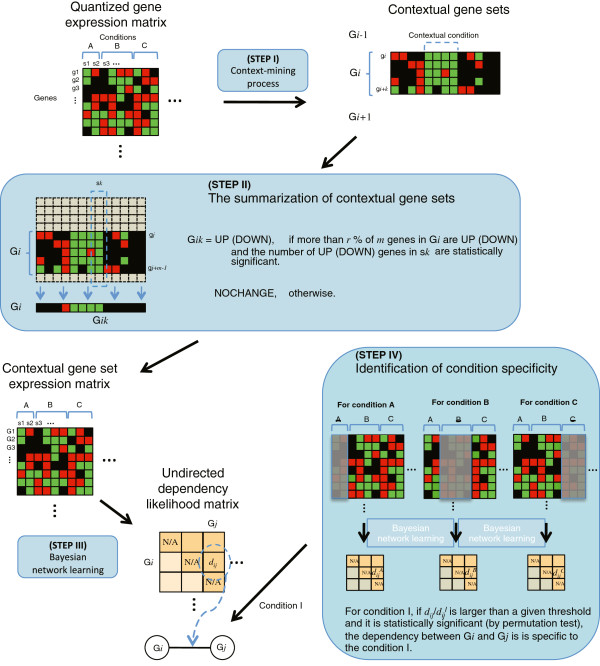
**The schematic overview of learning contextual gene set interaction networks and identifying condition specificity.** From the gene expression matrix, contextual gene sets are identified through the *context-mining* process. The expression values of genes in each contextual gene set for each sample are summarized into one major representative value, and a contextual gene set expression matrix is built as a result. Multiple Bayesian networks are learned from this matrix and their consensus network (undirected dependency likelihood matrix) is built while ignoring the direction of connections. For each condition, a subset of data is built by discarding the samples of the condition from the original data and a new dependency likelihood matrix is built from it. If the dependency likelihood of the interaction between **G**_*i*_ and **G**_*j*_ from all samples is significantly larger than the dependency likelihood from a data without a condition I, the interaction is specific to the condition I.

Learning contextual gene set interaction networks and identifying condition specificity involves several steps of data transformation, as illustrated in Figure 1, and is described further in the Methods section. It consists of four major steps: (STEP I) identifying contextual gene sets as basic functional modules, (STEP II) summarizing contextual gene sets to transform the data of genes to the data of contextual gene sets, (STEP III) learning contextual gene set interaction networks where each interaction represents dependency in expression (specific expression status of a gene set depends on the expression status of the other gene set) between two contextual gene sets, and (STEP IV) identifying condition specificity of each interaction. For the first step of identifying contextual gene sets, we define a contextual gene set as a set of genes that show consistent expression pattern under a biological context, i.e. a subset of samples. This is based on the assumption that once a biological context achieves a steady state, genes involved in the process show consistent transcriptional patterns under the biological context. Identifying contextual gene sets first requires the identification of samples where biological contexts are involved, and we use the *context-mining* algorithm [10,12] to find such contextual conditions, where a contextual condition is a subset of samples where groups of closely related coherent expression patterns are found. Under each contextual condition, sets of genes with similar over-expression or under-expression are identified as contextual gene sets (STEP I in Figure 1).

To infer networks of contextual gene sets, each contextual gene set is represented as a single variable. This requires that the original gene expression matrix needs to be transformed to a gene set expression matrix, where the value of a contextual gene set for a sample is a representative value of all genes in the contextual gene set. Expression values of genes in a contextual gene set for a sample are summarized to either UP or DOWN if the majority of the genes are over-expressed or under-expressed, and NOCHANGE value is given otherwise (STEP II). We are going to focus on the cases of statistically significant up-regulation or down-regulation, and most results from this study are from the cases of up or down-regulations.

A contextual gene set interaction network is learned from the summarized contextual gene set expression data, by evaluating the likelihood of dependency between each pair of contextual gene sets given all samples and building a connection if the dependency likelihood is larger than a given threshold (STEP III). Inference of interaction networks from the summarized data has a few advantages over traditional approach where all genes are used. Since the number of variables (nodes) is significantly smaller in this approach as all the genes in contextual gene set are aggregated to a single variable, the method suffers less in computational complexity, and thus it is subject to the curse of dimensionality to a lesser degree, leading to more reliable estimation of probability statistics on network models.

A resultant interaction between two contextual gene sets represents that there is a probabilistic dependency in their summarized expressions. Gene sets with dependency are expressed in coordinated manners, where the expression status of a gene set depends on the expression status of the other gene set. However, the effect to the dependency from the samples can be different for diverse conditions, as they can imply different activities of biological functions. Based on this idea, we identify condition-specific regions in the built network by measuring the effect from the samples of each condition on the likelihood of dependency. To measure the effect of a condition on a dependency, we evaluated the likelihood of the dependency without the samples of the condition and computed its difference with the original likelihood obtained using all available samples (STEP IV). If the original likelihood is significantly higher than the likelihood without the samples from the condition, it means that the samples under the condition have made significant contribution to the dependency. This implies that the dependency exists mainly due to the samples from the condition, thus it is declared as a condition-specific dependency.

### Example and advantage of identifying condition-specificity and contextual gene set

#### Example of identifying condition-specificity

**Figure 2:**
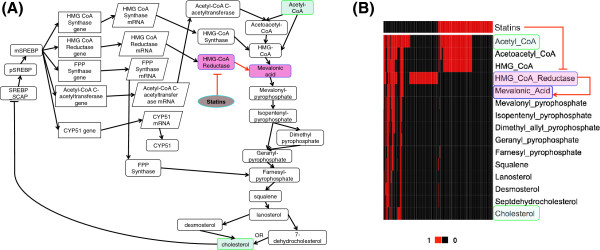
**The boolean network model of the cholesterol regulatory pathway. (A)** A network diagram of the model. All incoming connections into a node constitute AND logic except for cholesterol that has OR logic. A connection ending with a bar indicates NOT logic. The cholesterol synthesis pathway is shown from the precursor Acetyl-CoA to the final product cholesterol including feedback from cholesterol to *SREBP*-*SCAP*. Statins inhibits HMG-CoA reductase, and regulates the synthesis of cholesterol. After evaluating the specificity of each regulation to the statins perturbation, statins perturbation-specific regulations were colored with red. **(B)** A heat map of key intermediate products in the process of synthesizing cholesterol from Acetyl-CoA, together with the status of statins and HMG-CoA reductase. All 200 samples (100 without statins and 100 with statins) are shown.

One of key components of our approach is identifying condition-specificity of interactions in biological networks. To show the applicability of our method of identifying condition-specificity, we conducted a simulation experiment as an example. We used a boolean network model of cholesterol regulatory pathway [17] to generate synthetic data sets of two different conditions. The cholesterol regulatory pathway describes the synthesis of cholesterol from acetyl CoA. This process can be prohibited by drugs such as statins, and the boolean network model also includes the statins and its regulatory path. From the boolean network model of the cholesterol regulatory pathway, two synthetic data sets were generated with/without statins perturbation, where statins-perturbed data sets were generated by setting the state of statins to the value 1, which simulates a scenario that statins was given to block the synthesis of cholesterol, and the statins-free data were generated by setting the state of statins to the value 0, which simulates a normal scenario that cholesterol can be freely synthesized without the disturbance of statins (for the details of the synthetic data generation, see the Additional file 1). For each case of with/without statins perturbation, 100 samples were generated. The specificity to the statins perturbation and its statistical significance were evaluated for each regulatory relationship from the cholesterol pathway model, by using the method for identifying condition-specific interactions described in the Methods section. The same parameter values were used as described in the Methods section except for the number of permutations, as 1,000 permutations were used in this simulation example. Figure 2(A) illustrates a network diagram of the cholesterol regulatory pathway, and statins perturbation-specific regulations were highlighted with red color. As expected, the inhibitive regulation from statins to HMG-CoA reductase and its direct downstream regulation from HMG-CoA reductase to Mevalonic acid were identified as statins perturbation-specific regulations with statistical significance *P*<0.05. All other regulatory relationships did not pass the significance threshold *P*=0.05 (regulations represented with black connections), thus were not declared to be statins perturbation-specific. The presence of statins completely inhibits the existence of HMG-CoA reductase, and it stops HMG-CoA reductase from helping production of Mevalonic acid with the existence of HMG-CoA. This statins perturbation-specific regulations can be also seen from the heat map in Figure 2(B), which shows the boolean states of intermediate products that are produced in the process of synthesizing cholesterol from Acetyl-CoA. HMG-CoA reductase was inhibited with the presence of statins, and subsequently Mevalonic acid could not be produced regardless of the state of HMG-CoA. This heat map also shows that all the downstream products of Mevalonic acid do not exist with the presence of statins, either. However, their states are determined regardless of other elements once the state of Mevalonic acid is given, thus such later downstream regulations are not as specific to the presence of statins. This simulation example shows that the proposed method successfully identifies the regulatory paths of statins on the production of cholesterol, which are specifically affected by the condition of statins existence.

#### Advantage of contextual gene sets over biclusters

**Figure 3:**
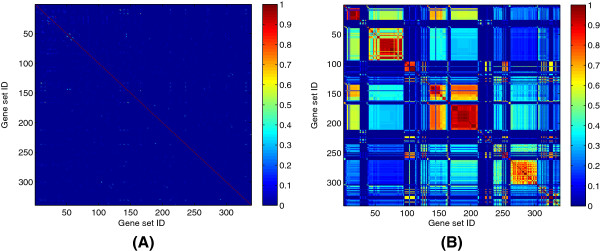
**Jaccard similarity heat maps of 339 gene sets from two different methods. (A)** Context-mining based method, **(B)** ISA.

We showed that the identification of condition-specificity in biological networks can be possible by the proposed method. However, applying that method to gene-level networks is computationally challenging when a target data covers a lot of genes, thus considering a set of genes as a functional module in the target network is a viable approach. We used the context-mining method to find contextual gene sets as such functional modules. Compared to the approach of context mining, conventional methods to cluster genes cannot identify gene sets that show coherent expression across a subset of samples, and thus final networks of gene sets will have only generic interactions across all samples, without any condition/subtype-specific interaction. Biclustering can be an alternative approach to find gene sets that show coherent expression under certain subset of samples, because it searches combinations of a subset of genes and a subset of samples, where the genes show similar patterns in the corresponding subset of samples. However, most biclustering methods lack the ability to group similar biclusters and often give significantly overlapping results. We compared our context mining based method and the Iterative Signature Biclustering Algorithm (ISA) [18] to see the overlap between their identified gene sets. From the refractory cancer data, 339 contextual gene sets were identified by our context mining based method, and the same number of biclusters were identified using ISA. The context mining based method gave significantly lower overlap between gene sets than ISA (Figure 3), thus using gene sets from the context mining based method will have less chance of inferring undesirable generic interactions between similar gene sets. This was confirmed by learning a gene set interaction network and identifying tissue-type specificity from the refractory cancer data, with gene sets from both methods. From the ISA gene sets, only 10 interactions were identified and none of them was determined to be tissue-specific, while 88 tissue-specific interactions were identified with the contextual gene sets, which will be described in the later sections.

### Contextual gene set interaction network from the refractory cancer gene expression data

#### Contextual gene set interaction network of refractory cancer with tissue type specificity

**Figure 4:**
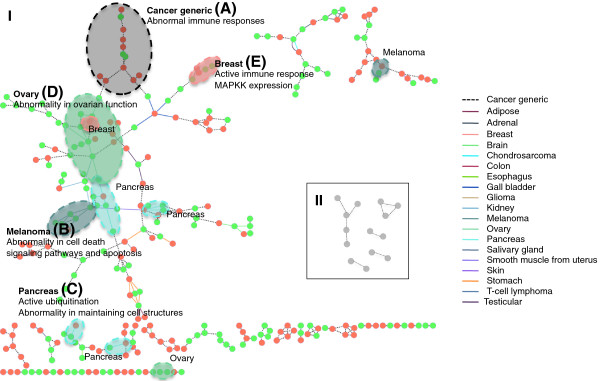
**The refractory cancer gene set interaction network annotated with the identified tissue type specificity.** (I) Network built with contextual gene sets. Only 278 contextual gene sets with at least one interaction are shown. The contextual gene sets under-expressed across their corresponding contextual conditions are colored with green and over-expressed contextual gene sets are colored with red. (II) Network built with gene sets from ISA biclusters. Only 14 bicluster gene sets are shown with 10 identified interactions. No tissue type specificity was found in this network.

We identified contextual gene sets and a network of the gene sets from the gene expression data of refractory cancer. From the gene expression data of 21,073 probes (from Agilent-011521 Human 1A Microarray G4110A) and 113 cancer patient samples of 32 different tissue types, 339 contextual gene sets were identified as functional modules. The gene expression data was summarized to an assortment of 339 contextual gene sets as shown in Additional file 2: Figure S1. By learning Bayesian networks from the summarized expression data, a dependency likelihood *d*_*ij*_ (=*d*_*ji*_) was evaluated for each pair of contextual gene sets **G**_*i*_ and **G**_*j*_, where 0≤*d*_*ij*_≤1. A contextual gene set interaction network of 285 interactions and 278 contextual gene sets was constructed by connecting gene sets with dependency likelihood larger than 0.5, which implies that they are more “likely” to exist. For each of 285 interactions in the contextual gene set interaction network, its specificity to each of 32 tissue types was evaluated and 88 interactions (31%) were identified to be tissue specific. The number of specific interactions for each tissue type is summarized in Table 1. Figure 4-I shows the contextual gene set interaction network and tissue type specificity, with contextual gene sets as nodes and interactions as edges. Edges have different styles and colors based on their tissue type specificity. In this network, tissue-specific interactions are associated with 19 tissue types and the other 13 tissue types did not have significant effect on the interactions. We also highlighted sub-networks enriched with interactions of certain tissue types by dotted ellipses after visual inspection. This result represents that the samples of different tissue types have different effect on the dependency between contextual gene sets. Compared to this contextual gene set interaction network, a network of ISA biclusters is also shown in Figure 4-II with the same style of tissue-specificity annotation. Only 10 interactions were identified and no tissue-specific interaction was found. This shows that our approach of using contextual gene sets can be more proper in identifying condition-specific gene set interactions than using conventional biclusters.

**Table 1:** The number of specific interactions for each tissue type from the refractory cancer data

	**Contextual gene set**	**ISA**
**All identified interactions**	**285**	**10**
Stomach	11	0
Pancreas	10	0
Melanoma	9	0
Adrenal	9	0
Ovary	8	0
Gall Bladder	7	0
Kidney	6	0
Breast	4	0
Brain	4	0
Testicular	4	0
Adipose tissue	3	0
Esophagus	2	0
Salivary Gland	2	0
Skin	2	0
Chondrosarcoma	2	0
Smooth muscle from uterus	2	0
Colon	1	0
T cell lymphoma	1	0
Glioma	1	0

#### Discrepancy in biological functions between cancer-generic and tissue-centric contextual gene sets

Each contextual gene set was annotated with Gene Ontology (GO) [19] terms of biological functions and pathways to further elucidate the meaning of the network. GATHER [20] was used to find associated annotations with statistical significance, with *P*=0.01 as a threshold of significance. To validate the discrepancy in functional meaning between cancer-generic interactions and tissue-specific interactions, the associated GO terms were compared between contextual gene sets with different types of interactions. From the contextual gene set interaction network in Figure 4-I, contextual gene sets with only interactions specific to a tissue type *T*_*k*_ were declared as *T*_*k*_-centric contextual gene sets. Similarly, contextual gene sets with only cancer-generic interactions were declared as cancer-generic contextual gene sets. Representing the set of GO terms associated with *T*_*k*_-centric contextual gene sets as **Annot**(*T*_*k*_), we compared the union of **Annot**(*T*_*k*_) from all tissue types to the GO terms associated with cancer-generic contextual gene sets, **Annot**(Cancer). The number of all tissue-centric contextual gene sets were 51 with $|\cup_{T_{k}}\mathrm{Annot}(T_{k})|=169$, while 175 contextual gene sets were cancer generic with |**Annot**(Cancer)|=413 (the rest 52 gene sets with mixed tissue type interactions were not considered in this process). $\cup_{T_{k}}\mathrm{Annot}(T_{k})$ and **Annot**(Cancer) have 93 common GO terms. Table 2 lists the most frequent GO terms associated with either only cancer-generic contextual gene sets or only tissue-centric contextual gene sets. Many GO terms associated with cancer-generic contextual gene sets are basic biological mechanisms and well-known biological functions related to general cancer, such as signal transduction, cell cycle and RNA processing. For tissue-specific functions, a few tissue-specific network regions will be discussed in detail in the later subsection with their functional annotations.

**Table 2:** Significantly associated GO terms to only cancer-generic or tissue-centric contextual gene sets

**GO terms relevant only to cancer-generic**		**GO terms relevant only to tissue-centric**	
**contextual gene sets**		**contextual gene sets**	
**GO term**	**Frequency**	**GO term**	**Frequency**
GO:0007166: cell surface receptor signal transduction	11	GO:0006163: purine nucleotide metabolism	2
GO:0007049: cell cycle	9	GO:0006164: purine nucleotide biosynthesis	2
GO:0006396: RNA processing	7	GO:0006629: lipid metabolism	2
GO:0008283: cell proliferation	7	GO:0009150: purine ribonucleotide metabolism	2
GO:0016070: RNA metabolism	7	GO:0009152: purine ribonucleotide biosynthesis	2
GO:0043207: response to external biotic stimulus	7	GO:0009259: ribonucleotide metabolism	2
GO:0000375: RNA splicing, via transesterification reactions	6	GO:0009260: ribonucleotide biosynthesis	2
GO:0000377: RNA splicing, via transesterification reactions	6	GO:0044255: cellular lipid metabolism	2
GO:0000398: nuclear mRNA splicing, via spliceosome	6	GO:0046148: pigment biosynthesis	2
GO:0006397: mRNA processing	6	GO:0000904: cellular morphogenesis	1
GO:0006959: humoral immune response	6	GO:0006099: tricarboxylic acid cycle	1
GO:0008380: RNA splicing	6	GO:0006119: oxidative phosphorylation	1
GO:0009613: response to pest, pathogen or parasite	6	GO:0006144: purine base metabolism	1
GO:0016071: mRNA metabolism	6	GO:0006188 : IMP biosynthesis	1
GO:0030333: antigen processing	6	GO:0006189: ’de novo’ IMP biosynthesis	1
GO:0000067: DNA replication and chromosome cycle	5	GO:0006510: ATP-dependent proteolysis	1
GO:0000075: cell cycle checkpoint	5	GO:0006554: lysine catabolism	1
GO:0006950: response to stress	5	GO:0006570: tyrosine metabolism	1
GO:0016064: humoral defense mechanism	5	GO:0006582: melanin metabolism	1
GO:0000279: M phase	4	GO:0006583: melanin biosynthesis from tyrosine	1

The frequency indicates the number of contextual gene sets associated with the corresponding GO term. Only 20 most frequent terms are shown for each case.

#### Cancer-generic network region

The region (A) in Figure 4-I is one example of a cancer-generic region. As this region includes many interactions that are not specific to certain tissue types, the expressions of the contextual gene sets within this region shows correlation across all tissue type samples. This region is mainly related to immune systems, as the 10 most statistically significant annotations are listed in Table 3. This can imply abnormal activity in immune mechanisms for the patients corresponding to the contextual conditions. Besides this example, the complete annotated network and the annotations of all contextual gene sets are given in the Additional file 3.

**Table 3:** Top 10 most significant annotations for 12 contextual gene sets of region (A), Figure 3

**MSigDB annotation (Source)**	***P***
Graft versus host disease (KEGG)	5.39E-08
Type I diabetes mellitus (KEGG)	9.56E-08
Natural killer cell mediated cytotoxicity (KEGG)	1.08E-07
Generation of second messenger molecules (REACTOME)	1.24E-07
Allograft rejection (KEGG)	1.69E-07
Viral myocarditis (KEGG)	9.33E-07
Translocation of ZAP70 to immunological synapse (REACTOME)	2.58E-06
Signaling in immune system (REACTOME)	3.97E-06
Leishmania infection (KEGG)	4.64E-06
Autoimmune thyroid disease (KEGG)	6.20E-06

#### Tissue-specific regions

The regions (B) – (E) in Figure 4-I are the examples of network regions where many tissue-specific interactions exist. For each region, the expression patterns of contextual gene sets show strong correlation for the corresponding tissue type samples (see Additional file 4: Figure S2(B-E)).

The melanoma-specific region (B) in Figure 4-I showed association with apoptosis. The five under-expressed contextual gene sets were related to abnormality in pigmentation, cell death signaling pathways and apoptosis. Individual contextual gene set shows further details in tissue-specific functional abnormalities. For example, **G**_122_ and **G**_223_ in the region (B) are related to the metabolism of nicotinamide and nicotinamide adenine dinucleotide (NAD ^+^) metabolism. Through these metabolisms, the coenzyme compound NAD ^+^ accepts or donates electrons in redox reactions [21] that play significant roles in releasing energy from nutrients by generating ATPs. This possible abnormal activity of energy generation can be related to the fact that melanoma (and also other cancers) is intensively positive in positron emission tomography (PET) scans due to their intense demand for energy, where tumor has up-regulated receptors that take in glucose and subsequently have high levels of glycolysis. One over-expressed contextual gene set in this region was related to GTP binding (*P*=1.71*E*−5) and guanyl nucleotide binding (*P*=1.37*E*−5), and this is supported by a report of the expression of small GTP-binding protein genes of the RAS family in melanoma [22].

**Figure 5:**
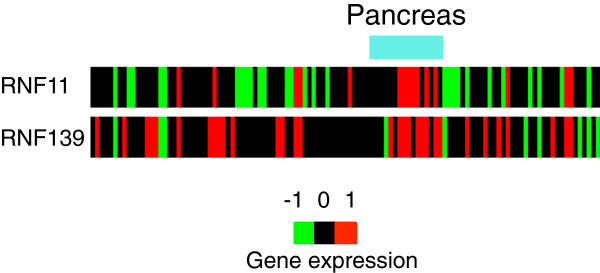
**View of *RNF11 * and *RNF139* expressions across the refractory cancer patient samples.** Gene expression values were transformed to log_2_ ratios compared to expressions from normal tissue samples.

For the pancreas-specific region (C) in Figure 4-I, the over-expressed **G**_198_ was associated with post-translational modification, such as ubiquitination. Ring finger proteins *RNF11* (7 out of 16 pancreas samples, *P*=0.001) and *RNF139* (9 out of 16 pancreas samples, *P*=0.0085) are over-expressed for several pancreas samples in **G**_198_ (Figure 5), and there is a report that the ubiquitin-editing enzyme *A20* downregulates *NF-κB* signaling in the presence of *RNF11*[23]. While the over-expressed **G**_198_ was related to post-translational modification with ubiquitination, the genes in the two under-expressed contextual gene sets (**G**_199_ and **G**_210_) were related to maintaining cell structures, which can facilitate cell motility and invasion.

The ovary-specific region (D) in Figure 4-I includes six contextual gene sets under-expressed in most of the ovarian samples, where they were associated with ovary-specific functional annotations such as reproduction and pregnancy. This can be related to the loss of normal ovarian function from the ovarian cancer patients. Besides the ovary-specific annotations, they are also related to the *β*-Arrestin pathway and the caspase mediated cleavage of cytoskeletal proteins. Arrestins can block G protein-mediated signaling, and redirect signaling to alternative G protein-independent pathways [24]. Regarding this annotation, there is a report that caspase mediated cleavage of cytoskeletal actin plays a positive role in the morphological changes of apoptosis [25].

### Contextual gene set interaction network from the GBM data of TCGA

#### Contextual gene set interaction network with phenotype/genotype specificity

**Figure 6:**
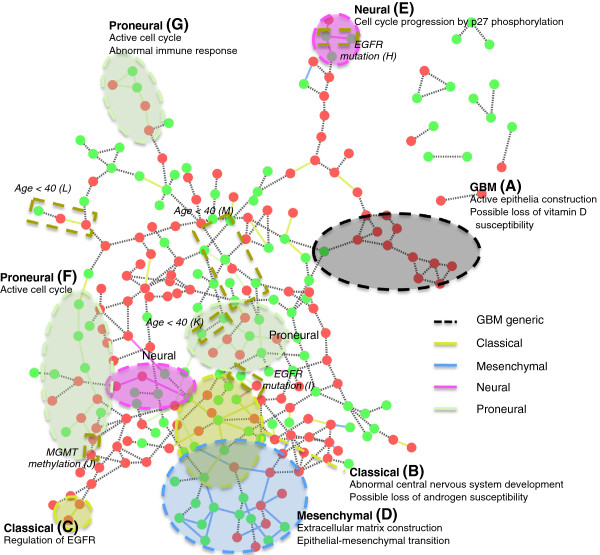
**The GBM contextual gene set interaction network annotated with the identified phenotype/genotype specificity.** Only 247 contextual gene sets with at least one interaction are shown. The contextual gene sets under-expressed across their corresponding contextual conditions are colored with green and over-expressed contextual gene sets are colored with red.

We also identified a contextual gene set interaction network from the gene expression data of TCGA GBM. From 202 GBM patient samples with four subtypes (Classical, Mesenchymal, Neural and Proneural) reported by Verhaak et al. [26], 316 contextual gene sets were identified. The gene expression data was summarized for the 316 contextual gene sets as shown in Additional file 5: Figure S3. Based on this summarized contextual gene set expression data, a contextual gene set interaction network (with 296 interactions and 247 contextual gene sets with at least one interaction) was built with the same methods and parameters applied to the case of the refractory cancer data analysis. For each interaction, its specificity to each of four subtypes was evaluated and 77 interactions (26%) were declared to be subtype specific. In addition to the four subtypes of GBM, specificities to the mutations of selected genes (*EGFR*, *NF1*, *PDGFRA*, *PIK3CA*, *PIK3R1* and *TP53*), the methylation of *MGMT*, and age <40 were also evaluated. The number of specific interactions for each subtype and condition is given in Table 4. Figure 6 shows the contextual gene set interaction network with identified phenotype/genotype specificity. After visual inspection, regions enriched with interactions of certain subtypes were highlighted with dotted ellipses. Among the tested genetic mutations, only *EGFR* mutation showed associated interactions.

**Table 4:** The number of specific interactions for each GBM subtype and sample condition

**Subtype/Condition**	**Number of specific interactions**
Classical	24
Mesenchymal	20
Neural	8
Proneural	24
*EGFR* mutation	2
*MGMT* methylation	1
Age < 4\0	7

Among the investigated conditions, conditions with no specific interaction are not shown.

#### Functional difference between GBM-generic and subtype-centric contextual gene sets

Each contextual gene set was annotated with GO terms of biological functions and pathways, and significantly associated GO terms were compared for the contextual gene sets that have different types of interactions. From the GBM contextual gene set interaction network in Figure 6, the annotations of subtype *T*_*k*_-centric contextual gene sets were compared to the annotations of GBM-generic contextual gene sets. Table 5 lists the number of each subtype-centric contextual gene sets with their associated annotation terms. From Table 5, all four (100%) GO terms associated with Neural-centric contextual gene sets and 31 out of 35 (88.6%) GO terms associated with Proneural-centric contextual gene sets were also associated with GBM-generic contextual gene sets. This can imply the closeness of the abnormalities in Neural and Proneural subtypes to GBM-generic abnormalities. Compared to the cases of Neural and Proneural subtypes, none or few of the GO terms from Classical (none out of four, 0%) and Mesenchymal (6 out of 34, 17.6%) subtypes were overlapping with the GO terms associated with GBM-generic contextual gene sets, which can imply these two subtypes are more differentiated forms of GBM than Neural and Proneural subtypes. These findings of Classical and Mesenchymal subtypes being more differentiated GBM are consistent with the results of a previous GBM study [26].

**Table 5:** The number of subtype-centric contextual gene sets and associated annotation terms

***T***_***k***_	***T***_***k***_**-centric gene sets**	**Genes**	**|Annot**(***T***_***k***_)**|**	**|Annot**(***T***_***k***_) ∩ **Annot**(***GBM*)| (Overlap %)**
Classical	6	317	4	0 (0%)
Mesenchymal	8	564	34	6 (17.6%)
Neural	4	577	4	4 (100%)
Proneural	14	1,408	35	31 (88.6%)

#### Comparison of contextual gene sets with other gene signatures of GBM subtypes

We compared the genes in subtype-centric contextual gene sets with the GBM subtype signature genes reported by Verhaak et al. [26]. Table 6 lists the number of over/under-expressed genes for each subtype, from two methods. Subtype-centric contextual gene sets have completely different list of genes compared to the gene signatures reported by Verhaak et al., as there was no overlap across all subtypes. This significant difference is due to the different approaches of identifying genes from two methods. The GBM subtype signature genes by Verhaak et al. were identified in two steps – they first used a consensus clustering to group the patient samples into four stable clusters, and then, differentially over-expressed genes in each subtype were identified using the combination of significance analysis of microarrays (SAM), receiver operating characteristic (ROC) methods and ClaNC, a nearest centroid-based classifier [26]. In contrast, contextual gene sets were identified with more focus on biological processes rather than differentially expressed signatures of subtypes. Genes in each contextual gene set are grouped as they show statistical interactions amongst them based on their expression levels, and each subtype-centric contextual gene set has its interactions strongly associated with a specific subtype. Thus, subtype-specific contextual gene sets represent genes whose interactions are strongly associated with biological processes underlying certain specific subtypes, rather than gene signatures for certain subtypes. In addition, as some genes may function in multiple different biological processes, a gene may belong to multiple contextual gene sets, and this is another discrepancy between contextual gene sets and subtype gene signatures.

**Table 6:** Comparison between GBM subtype-centric contextual gene sets and GBM subtype signature genes reported by Verhaak et al. [26]

**Gene set**	**Classical**		**Mesenchymal**		**Neural**		**Proneural**	
	**UP**	**DOWN**	**UP**	**DOWN**	**UP**	**DOWN**	**UP**	**DOWN**
Subtype-centric contextual gene set	309	8	27	537	362	215	612	796
Verhaak et al.	162	0	216	0	129	0	178	0
Overlap	0		0		0		0	

Table lists the number of over-expressed genes (UP) and under-expressed genes (DOWN).

The identified contextual gene sets were also compared to the result of Gene Set Enrichment Analysis (GSEA) on MSigDB [27], which is a method to identify differentially expressed gene sets for each condition, based on prior knowledge. For GSEA, the standardized gene expression data was used without quantization. GSEA analysis was done by comparing “samples of one subtype versus the rest of the samples” for each subtype, to identify subtype-specific gene sets. Out of 2,101 gene sets of canonical pathway gene sets and GO gene sets of biological process and molecular function from MSigDB, 2,067 gene sets (98.4%) with up to 500 genes were tested using GSEA. In running GSEA for each gene set, 1,000 permutations were applied. From the result, *P* values were FDR-corrected using Benjamini and Hochberg’s method, and FDR-corrected *P*=0.05 was used for statistical significance. Table 7 lists the number of identified gene sets by using two methods, where GBM-generic or each subtype-centric gene sets are shown for contextual gene sets, and subtype-specific gene sets are shown for GSEA. Our method could identify GBM-generic contextual gene sets as well as multi-type contextual gene sets, which show interactions with other contextual gene sets across multiple subtypes of GBM. Such types of gene sets cannot be identified using conventional GSEA, which works in a supervised manner based on the given subtype information. Another finding is that GSEA returns very biased number of gene sets between subtypes, where the Mesenchymal subtype dominates the number of findings. A possible hypothesis of GSEA returning biased results for Mesenchymal is that Mesenchymal is a most differentiated form of GBM (physiologically or genotypically) [26] and many genes are differentially expressed in Mesenchymal compared to other subtypes. Compared to GSEA, our method gives less biased results to a specific subtype, and this is because subtype-centric contextual gene sets are identified based on subtype-specific interactions. As subtype-specific interactions are focused on consistent interactions instead of consistent expression levels, they are relatively free from the bias of consistent differential expression in each subtype.

**Table 7:** Comparison between contextual gene sets and MSigDB gene sets identified with GSEA

	**GBM-generic**	**Classical**	**Mesenchymal**	**Neural**	**Proneural**	**Multi-type**
Contextual gene set	144	6	8	4	14	71
GSEA	N/A	1	245	6	3	N/A

The number of gene sets are listed. For the contextual gene sets, GBM-generic or each subtype-centric contextual gene sets are shown. *Multi-type* indicates gene sets with various subtype-specific interactions.

#### GBM-generic network region

The network region (A) in Figure 6 is one example of a GBM-generic region with 12 contextual gene sets in it. The heat map (Additional file 6: Figure S4(A)) of the contextual gene sets show that their expressions are closely correlated across all patient samples, making the interactions among these contextual gene sets be GBM-generic. From the associated annotations of the over-expressed contextual gene sets in their corresponding contextual conditions, this network region was mainly related to the tight junction and the intercellular adhesion, which occur in epithelia and brain endothelia. Also by considering other annotations such as epithelial cell differentiation and morphogenesis of an epithelium, this network component can represent active epithelia construction in GBM, which can imply active blood vessel construction. For the MSigDB annotations of the under-expressed contextual gene set, the presence of a transcriptional start site motif was statistically significant, which matches annotations for vitamin D receptor *VDR*. Considering the function of vitamin D killing GBM cells [28], a possible hypothesis is that the loss of vitamin D susceptibility in GBM patients can be related to the low activities of genes targeted by *VDR*, while the main cause of such low activities remains for further studies.

#### GBM subtype-specific regions

**Figure 7:**
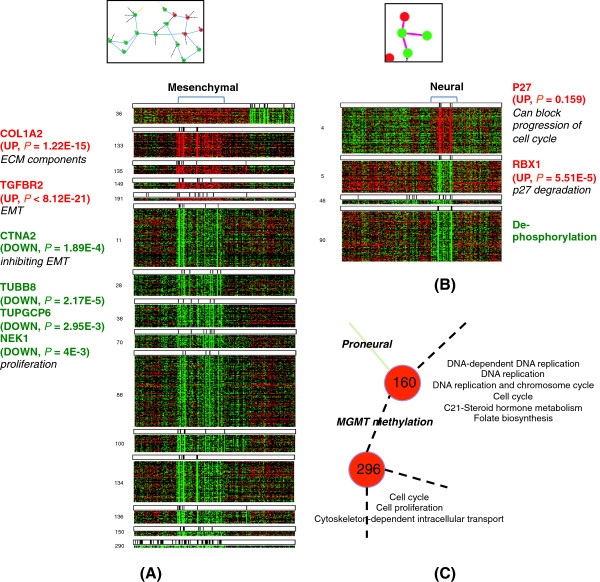
**Closer look on selected condition-specific regions from the GBM contextual gene set interaction network. (A)** Heat maps of the Mesenchymal-specific region **(D)**, with a brief summary of its relatedness to Mesenchymal features. **(B)** Heat maps of the Neural-specific region **(E)**, where this region can represent a cell cycle progression by p27 phosphorylation. **(C)***MGMT* methylation-specific region **(J)**, where *MGMT* methylation is related to the repair of damaged DNA in the process of cell cycle.

The regions (B) – (G) in Figure 6 show the examples of subtype-specific regions for four subtypes of GBM. In each example, the expression patterns of contextual gene sets show strong correlation for the corresponding subtype samples (see Figure 7(A) and (B), and Additional file 6: Figure S4).

The Classial-specific region (B) in Figure 6 includes six contextual gene sets, where one is over-expressed and the other five are under-expressed across Classical samples. From the MSigDB analysis, one of the significant annotations related to the over-expressed contextual gene set is a transcriptional start site motif that matches annotation for a member of *ETS* oncogene family, *ELK1*, which is involved in pro-apoptotic and pro-differentiation in neuronal cells [29], and *MAPK*-*ELK1* signaling pathway that contributes to cell survival [30]. This implies that genes targeted by *ELK1* are over-expressed and *ELK1* is enabling many of its downstream genes. The genes in the under-expressed contextual gene sets were related to central nervous system development (*P*=2.58*E*−4), which can imply abnormalities in neural system development. A transcriptional start site motif was also related to the under-expressed contextual gene sets, which matches annotation for the androgen receptor *AR*. It has been reported that *AR* was detected in a higher proportion of gliomas [31], while there was a suggestion that the proliferative effect of GBM may not be through the activation of *AR*[32]. However, there are reports of activated *AR* enhancing susceptibility of GBM cells to chemotherapeutics and radiation therapy [33], and promoting cell death [34]. These reports suggest that there is a certain group of patients with low *AR* activity, where the activation of *AR* combined with other therapeutics can be a potential treatment for such patients. The Classical-specific region (C) reveals the presence of *N-Myc* over-expression, which directly regulates a number of genes associated with the classical phenotype gene signature including *EGFR*[35].

For the Mesenchymal-specific region (D) in Figure 6, five over-expressed contextual gene sets for Mesenchymal samples are related to cell surface interactions (integrin cell surface interactions, *P*=7.3*E*−5) and several signaling pathways related to cancer. We could see up-regulation of Collagen I, IV ECM components (*COL1A2* up-regulation in 33 out of 58 Mesenchymal samples, *P*=1.22*E*−15; *COL4A5* up-regulation in 10 Mesenchymal samples, *P*=0.1069) that signify increased ECM production. TGF beta receptor II is also up-regulated (46 out of 58 Mesenchymal samples, *P*<8.12*E*−21), which is associated with epithelial-mesenchymal transition (EMT). Jak-STAT signaling pathway was related, too (*P*=1.84*E*−4), with *PIK3CD* and *JAK2* up-regulation. Genes involved in integrin cell surface interactions (*COL1A2*, *ITGA11*, *RAP1B*, *COL4A5* and *APBB1IP*) were also included and these can lead to *MAPK* signaling [36]. The other 10 contextual gene sets under-expressed in many Mesenchymal samples include down-regulated *CTNNA2* (down-regulation in 18 of 58 Mesenchymal samples, *P*=1.89*E*−4), where *CTNNA2* is known to control the stability of dendritic spines and synaptic contacts [37]. This suggests that EMT in GBM also accompanies the low activity of *CTNNA2* and resulting instability of neuronal cell-cell structures. These gene sets are also related to microtubule cytoskeleton organization and biogenesis (*P*=1*E*−5). Besides, we could observe the down-regulation of tubulins (*TUBB8* down-regulation in 30 of 58 Mesenchymal samples, *P*=2.17*E*−5; *TUBGCP6* down-regulation in 23 of 58 Mesenchymal samples, *P*=2.95*E*−3) and *NEK1* (25 of 58 Mesenchymal samples, *P*=4*E*−3) and *NEK11* (16 of 58 Mesenchymal samples, *P*=0.263), which are components of proliferation, and it suggests a hypothesis that proliferation activity can be low in Mesenchymal cells since their high migratory behavior. A brief summary and a heat map of gene expressions of this region is shown in Figure 7(A), and the findings mentioned above can imply active EMT, abnormalities in maintaining cell structures, and low proliferative activity, which well fit the characteristics of the Mesenchymal subtype.

The Neural-specific region (E) in Figure 6 can represent the cell cycle progression by p27 phosphorylation. The over-expressed **G**_4_ includes p27 (12 of 33 Neural samples, *P*=0.159) and *RBX1* (16 of 33 Neural samples, *P*=5.51*E*−5), where p27 can block progression of cell cycle [38]. However, F box protein binds with phosphorylated p27 with the involvement of *RBX1*, causes p27 degradation and cell cycle progression [39]. Accordingly, the three under-expressed contextual gene sets were related to dephosphorylation (*P*=2.45*E*−3). A summary and a heat map of this region is also shown in Figure 7(B).

The Proneural-specific region (F) in Figure 6 includes over-expressed contextual gene sets that are related to cell cycle, and under-expressed contextual gene sets related to homeostatic processes. The Proneural-specific region (G) also shows active cell cycle processes, and degraded immune responses.

#### Other genotype/phenotype-specific regions

In addition to the four subtypes of GBM, other condition-specific interactions were also identified. Among genetic mutations, only *EGFR* mutation has associated interactions in regions (H) and (I). The condition of ages <40 is associated with regions (K), (L) and (M). An interesting result is the region (J) associated with the methylation of *MGMT* (Figure 7(C)). In the region (J), the two over-expressed contextual gene sets, which are connected with a *MGMT* methylation-specific interaction, were related to cell cycle, especially DNA replication and checkpoint (DNA dependent DNA replication with *P*=7.84*E*−10, mitotic cell cycle with *P*=3.47*E*−9 and mitotic M-M/G1 phases with *P*=1.59*E*−7). This specificity of *MGMT* methylation to the DNA replication and checkpoint in cell cycle is evident, by considering that *MGMT* is involved in the process of repairing damaged DNA during the replication process. The expression of *MGMT* could have been disturbed by methylation and eventually lost its function in the cell cycle checkpoint. The review by Casorelli et al. [40] also covers the role of the *MGMT* repair protein in cancer.

## Conclusions

High heterogeneity in cancer has been evident since early studies, and approaches to reveal such heterogeneity embedded in genomic profiling data are showing promising results. In this work, we used a novel method of measuring the effect from expression samples of certain conditions on the components in contextual gene set interaction networks to identify condition-specificity. In addition to the simulation experiment, two cancer data sets were analyzed to support the validity of identifying condition-specificity, which are the refractory cancer data with 32 tissue types and TCGA GBM data with four subtypes. Contextual gene set interaction networks were built with tissue/phenotype/genotype-specificities. The resultant contextual gene set interaction networks with specificities showed different interaction patterns across conditions, and they provided new hypotheses as well as consistency with previous studies. Bayesian network learning was used in this work to more correctly estimate the likelihood of dependency, but simpler measures such as correlation or mutual information can be also used for the same formulation. Thorough analysis will follow this study for condition-specific interactions and related contextual gene sets to further analyze biological mechanisms of condition specificity in cancer. We are also developing methods to classify new patient samples based on the identified contextual features. Specifically, the analysis results of TCGA GBM data in this study are being considered to subtype additional GBM patient samples from TCGA as well as GBM xenograft models with drug response information. Such results of validation using independent GBM samples will be further discussed in future studies.

## Methods

### The refractory cancer and TCGA-GBM gene expression data

For the case of analyzing the refractory cancer data [16], gene expression data of 21,073 probes (from Agilent-011521 Human 1A Microarray G4110A) and 113 patient samples (32 different types of refractory cancer) were used in this study. The consenting of the patients involved has been performed as described in [16]. The patients ranged in ages of 27 - 75 and there was no juvenile. For each tumor type, its (normal) tissue of origin was used as a baseline and the ratio of the tumor to its tissue of origin was computed, using a statistical model [41], and the ratio value was quantized to three discrete values of UP, DOWN and NOCHANGE with two-fold change as threshold. This two-fold change threshold was to ensure quantized values to represent minimal changes that can be reliably reproducible. The distribution of the 113 samples among different cancer tumor types is listed in the Additional file 1: Table S1.

As for the GBM study, we downloaded the GBM gene expression data from The Cancer Genome Atlas (http://cancergenome.nih.gov/). Among those, 202 samples with four known subtypes (54 Classical, 58 Mesenchymal, 33 Neural and 57 Proneural [26]) were used in this study.

Since we only used gene-level summary values for TCGA-GBM data, we used a heuristic approach to discretize the data for our analysis. The expression of 17,814 genes in the GBM samples were converted to z-scores using 10 normal samples as a reference. The standardized expression values were quantized to three levels of UP, DOWN and NOCHANGE by using one standard deviation as a threshold. Higher threshold resulted in too many NOCHANGE values, which led to less informative data for the analysis. The detailed sample information is available in the Additional file 3.

### Identifying contextual gene sets

We define a contextual gene set as a set of genes that show consistent expression pattern under a biological context. This is based on the assumption that once a biological context reaches a steady state, genes involved in the process show consistent patterns under the biological context. It requires the identification of subsets of samples, which are representations of biological contexts. We use the *context-mining* algorithm [10,12] to find contextual conditions as such samples, where a contextual condition is a subset of samples that have groups of closely related coherent expression patterns (such patterns are called *context-motifs* in the algorithm). In the process of context-mining, two consistency statistics *conditioning* (*δ*) and *crosstalk* (*η*) are used to find context-motifs, and a permutation test is applied to check their statistical significance. In our study, *δ*=0.1, *η*=0.3 and the significance *P*<0.05 (Benjamini and Hochberg corrected) were used. With the graphical representation of context-motifs, the Markov cluster (MCL) algorithm [42] is used to cluster closely related context motifs into biological contexts. The *inflation* parameter was set to 2 in our study, as suggested by the developer of MCL. A representative set of samples from each identified context is determined as a contextual condition using the sample association score (SAS) [43], and we used SAS <0.5 in our study. For each contextual condition, consistency statistics *δ* and *η* were used again to find contextual gene sets that include genes with consistent over-expression or under-expression. The same *δ*=0.1, *η*=0.3 and *P*<0.05 were used in this step.

### Summarizing gene sets

To infer networks of contextual gene sets, each contextual gene set needs to be represented as a single variable as most network models assume each node as a single random variable. For each sample *s*_*k*_, the expression values of *m* genes in a contextual gene set **G**_*i*_ is summarized to a single representative value *G*_*ik*_, where *G*_*ik*_ is UP if more than *r* % of the genes in **G**_*i*_ are over-expressed with statistical significance of a hypergeometric *P* lower than a given threshold (vice versa for the case of DOWN). Otherwise, *G*_*ik*_ is given a value of NOCHANGE. In this study, *r*=50*%* and a *P* threshold 0.05 were used.

### Learning contextual gene set interaction networks

A contextual gene set interaction network is built from **S**_*U*_, which is a set of all samples after the expression summarization of the original gene expression matrix **D**, by computing the likelihood of dependency *d*_*ij*_= Pr(**G**_*i*_↔**G**_*j*_|**S**_*U*_) (=*d*_*ji*_) between each pair of contextual gene sets **G**_*i*_ and **G**_*j*_. **G**_*i*_↔**G**_*j*_ is a connection between two contextual gene sets **G**_*i*_ and **G**_*j*_ in any direction. We used the Bayesian network model to identify the dependency between contextual gene sets, and the BANJO software (http://www.cs.duke.edu/âˆ¼amink/software/banjo/) was used to learn Bayesian networks. From *R* independent runs of BANJO with **S**_*U*_, *d*_*ij*_ was computed as a frequency of the undirected connection **G**_*i*_↔**G**_*j*_ as follows:

(1) $$d_{\text{ij}}=\frac{\sum_{k=1}^{R}F(BN_{k},G_{i}↔G_{j})}{R},$$

where *BN*_*k*_ is a Bayesian network structure from *k*th run of BANJO, and *F* is a function that returns 1 if *BN*_*k*_ has **G**_*i*_↔**G**_*j*_, or 0 otherwise. The direction of connections in the Bayesian networks was ignored as we consider either direction of a connection to represent the same existence of dependency between two contextual gene sets. If *d*_*ij*_ is larger than a given threshold *d*_*θ*_, we declared that a dependency exists between **G**_*i*_ and **G**_*j*_. In our study, we used *R*=1,024 and *d*_*θ*_=0.5.

### Identifying condition-specific network components

Our approach to identify the specificity of a dependency relationship to a condition is measuring the effect by the samples of a condition on the likelihood of the dependency. When $S_{T_{k}}$ is a set of samples of a condition *T*_*k*_, the amount of effect *γ* by $S_{T_{k}}$ on a dependency relationship **G**_*i*_↔**G**_*j*_ is defined by the relative ratio of the dependency likelihood with **S**_*U*_ to the likelihood with $S_{U}-S_{T_{k}}$. More specifically, *γ* can be defined as follows:

(2) $$\gamma (G_{i}↔G_{j};S_{U},S_{T_{k}})=\frac{d_{\text{ij}}}{d_{\text{ij}}^{T_{k}}},$$

where $d_{\text{ij}}^{T_{k}}=\mathrm{Pr}(G_{i}↔G_{j}|S_{U}-S_{T_{k}})$. One characteristic of *γ* is that $\gamma (G_{i}↔G_{j};S_{U},S_{T_{k}})$ is proportional to $\mathrm{Pr}(G_{i}↔G_{j}|S_{T_{k}})$, which is a conventional measure for the relevance of **G**_*i*_↔**G**_*j*_ to the type *T*_*k*_.

#### 

**Theorem 1.** Once $S_{T_{k}}$ is given, $\gamma (G_{i}↔G_{j};S_{U},S_{T_{k}})$ is proportional to $\mathrm{Pr}(G_{i}↔G_{j}|S_{T_{k}})$.

#### 

*Proof.* With the Bayes’ theorem,

(3) $$\gamma (G_{i}↔G_{j};S_{U},S_{T_{k}})=\frac{\mathrm{Pr}(G_{i}↔G_{j}|S_{U})}{\mathrm{Pr}(G_{i}↔G_{j}|S_{U}-S_{T_{k}})}$$

(4) $$\begin{array}{lll} = & \frac{\mathrm{Pr}(S_{U}|G_{i}↔G_{j})\mathrm{Pr}(G_{i}↔G_{j})}{\mathrm{Pr}(S_{U})}\;\\ & \times \frac{\mathrm{Pr}(S_{U}-S_{T_{k}})}{\mathrm{Pr}(S_{U}-S_{T_{k}}|G_{i}↔G_{j})\mathrm{Pr}(G_{i}↔G_{j})}\; & \; \end{array}$$

(5) $$=\frac{\mathrm{Pr}(S_{U}|G_{i}↔G_{j})}{\mathrm{Pr}(S_{U}-S_{T_{k}}|G_{i}↔G_{j})}\times \frac{\mathrm{Pr}(S_{U}-S_{T_{k}})}{\mathrm{Pr}(S_{U})}$$

By assuming independence in observing each sample given **G**_*i*_↔**G**_*j*_,

(6) $$\;\gamma (G_{i}↔G_{j};S_{U},S_{T_{k}})=\mathrm{Pr}(S_{T_{k}}|G_{i}↔G_{j})\times \frac{\mathrm{Pr}(S_{U}-S_{T_{k}})}{\mathrm{Pr}(S_{U})}$$

With the Bayes’ theorem,

(7) $$\mathrm{Pr}(G_{i}↔G_{j}|S_{T_{k}})=\frac{\mathrm{Pr}(S_{T_{k}}|G_{i}↔G_{j})\mathrm{Pr}(G_{i}↔G_{j})}{\mathrm{Pr}(S_{T_{k}})}$$

Thus,

(8) $$\mathrm{Pr}(S_{T_{k}}|G_{i}↔G_{j})=\frac{\mathrm{Pr}(S_{T_{k}})}{\mathrm{Pr}(G_{i}↔G_{j})}\times \mathrm{Pr}(G_{i}↔G_{j}|S_{T_{k}})$$

From equations (6) and (8),

(9) $$\begin{array}{lll} \;\gamma (G_{i}↔G_{j};S_{U},S_{T_{k}})=\frac{\mathrm{Pr}(S_{T_{k}})}{\mathrm{Pr}(G_{i}↔G_{j})} & \times \mathrm{Pr}(G_{i}↔G_{j}|S_{T_{k}})\;\\ & \times \frac{\mathrm{Pr}(S_{U}-S_{T_{k}})}{\mathrm{Pr}(S_{U})}\; & \; \end{array}$$

(10) $$\;=\frac{1}{\mathrm{Pr}(G_{i}↔G_{j})}\times \frac{\mathrm{Pr}(S_{U}-S_{T_{k}})\mathrm{Pr}(S_{T_{k}})}{\mathrm{Pr}(S_{U})}\times \mathrm{Pr}(G_{i}↔G_{j}|S_{T_{k}})$$

By assuming a uniform prior for Pr(**G**_*i*_↔**G**_*j*_), $\frac{1}{\mathrm{Pr}(G_{i}↔G_{j})}$ is a constant. And once $S_{T_{k}}$ is given, $\frac{\mathrm{Pr}(S_{U}-S_{T_{k}})\mathrm{Pr}(S_{T_{k}})}{\mathrm{Pr}(S_{U})}$ is also a constant. Thus, the equation (10) can be written as follows:

(11) $$\gamma (G_{i}↔G;S_{U},S_{T_{k}})=C\times \mathrm{Pr}(G_{i}↔G_{j}|S_{T_{k}}),$$

where *C* is a constant. Therefore,

(12) $$\gamma (G_{i}↔G;S_{U},S_{T_{k}})\propto \mathrm{Pr}(G_{i}↔G_{j}|S_{T_{k}})$$

□

The benefit of this characteristic is that *γ* can be used instead of the conventional measure $\mathrm{Pr}(G_{i}↔G_{j}|S_{T_{k}})$, especially when the direct measurement of $\mathrm{Pr}(G_{i}↔G_{j}|S_{T_{k}})$ can be unreliable due to the limited number of samples for each condition, which is the case of many biological applications.

To measure the statistical significance of $\gamma (G_{i}↔G_{j};S_{U},S_{T_{k}})$, a permutation test is done by using $S_{T_{k}}^{r}$ instead of $S_{T_{k}}$, which is built by randomly selecting $\left|S_{T_{k}}\right|$ samples from **S**_*U*_. If *H* out of *M* permutations gave *γ* greater than or equal to $\gamma (G_{i}↔G_{j};S_{U},S_{T_{k}})$, the statistical significance *P* of $\gamma (G_{i}↔G_{j};S_{U},S_{T_{k}})$ is *H*/*M*. When $\gamma (G_{i}↔G_{j};S_{U},S_{T_{k}})$ is larger than a given threshold *γ*_*θ*_ and its *P* is lower than a threshold, **G**_*i*_↔**G**_*j*_ is declared to be specific to the condition *T*_*k*_. In our study, we limited *M* to 100 for each significance test of *γ* due to the computational cost of repeating Bayesian network learning many times for each permutation. *γ*_*θ*_=2, which indicates two-fold or higher increase of dependency relationship by adding the sample of the condition *T*_*k*_, and *P*=0.05 were used as threshold values.

### Annotation of gene sets

GATHER [20] was used to identify GO terms that are associated to each contextual gene set with statistical significance, where the terms from molecular function and biological process categories were considered. *P*=0.01 was used as a significance threshold. For annotating specific regions in a contextual gene set interaction network, we computed the overlap of genes (all, over-expressed, or under-expressed) in a region with the pathway gene sets in MSigDB [27], and its statistical significance was evaluated with a hypergeometric *P*. After the false discovery rate (FDR) correction of *P* values using Benjamini and Hochberg’s method, FDR-corrected *P*=0.01 was used as a significance threshold.

## Abbreviations

CML: Chronic myelogenous leukemia; TCGA: The Cancer Genome Atlas; GBM: glioblastoma multiforme; ISA: Iterative Signature Biclustering Algorithm; GO: Gene Ontology; NAD: nicotinamide adenine dinucleotide; PET: positive in positron emission tomography; EMT: epithelial-mesenchymal transition; MCL: Markov cluster; SAS: sample association score

## Competing interests

The authors declare that they have no competing interests.

## Authors’ contributions

SJ and SK designed the research. SJ conceived and performed the data analysis and modeling with the aid of MV and SK. SJ and JK analyzed the results with contributions from DH, M Berens, M Bittner and SK. SJ and MV wrote the article with contributions from JK, M Berens, M Bittner and SK. All authors read and approved the final manuscript.

## Supplementary Material

Additional file 1 supplementary_information.pdf, 507K. Click here for file

Additional file 2 **Figure S1.** The summarized gene set expression data of refractory cancer patients. Click here for file

Additional file 3 supplementary_data.xls, 3406K. Click here for file

Additional file 4 **Figure S2.** The heat maps of cancer-generic region and tissue specific regions from the refractory cancer contextual gene set interaction network. Click here for file

Additional file 5 **Figure S3.** The summarized gene set expression data of GBM samples from TCGA. Click here for file

Additional file 6 **Figure S4.** The heat maps of GBM-generic region and GBM subtype-specific regions from the TCGA-GBM contextual gene set interaction network. Click here for file
