# Supplementary material for: Learning contextual gene set interaction networks of cancer with condition specificity
Source: BMC Genomics. 2013 Feb 19;14:110. doi: 10.1186/1471-2164-14-110 (PMC3644282; doi:10.1186/1471-2164-14-110)
Supplement: Additional file 1 — supplementary_information.pdf, 507K. [file 1471-2164-14-110-S1.pdf]

Supplementary Tables

Table S1

The number of samples for each tissue type in the refractory cancer data.

Tissue type	Number of samples	Tissue type	Number of samples
Melanoma	17	Pancreas	16
Ovary	15	Breast	9
Adrenal	5	Colon	5
Kidney	5	Brain	4
Adipose tissue	3	Bladder	3
Gall Bladder	3	Adenoid Cyst Saliv Gl	2
Esophagus	2	Lung	2
Salivary Gland	2	Skin	2
Stomach	2	T cell lymphoma	2
Cartilage	1	Cervical	1
Chondrosarcoma	1	Gastric	1
Glioma	1	Ileum	1
Lymphoma	1	Monocytes	1
Rhabdomyosarcoma	1	Skeletal muscle	1
Smooth muscle from uterus	1	Synovial cell sarcoma	1
Testicular	1	Thyroid	1

Supplementary information I – Synthetic data generation from the Boolean network model of the cholesterol pathway

We assume that only the observation on steady states of the pathway is possible for data sampling. The proper way of sampling from steady states should be running the Boolean network from every possible initial state until an attractor is encountered and identifying the distribution of steady states based on the statistics of attractors. However, the Boolean network model of cholesterol regulatory pathway has 33 variables and there are total $2^{33} \approx 8.59 \times 10^{9}$ possible states, thus it is infeasible to trace the dynamics from all initial states. For this reason, we use an empirical approach to decide the sampling probability of each state.

Let us suppose that a state *s* belongs to an attractor *A*, where an attractor is a subset of states from which a Boolean network cannot go to any other state than the ones in the attractor. The probability of observing *s* and *A* is as follows:

$$P(s, A) = P(s|A)P(A)$$

Because the observation of *s* implies the observation of *A*, $P(s, A) = P(s)$. As a result, the probability of observing *s* becomes as follows:

$$P(s) = P(s|A)P(A)$$

This can be interpreted as the observation of *s* is done by first observing *A* and then observing *s* among the states in *A*.

In this simulation, we take the following scenario of observing the steady states of the cholesterol regulatory pathway in two different conditions – the absence of statins and the existence of statins.

1. Observing a steady state from the pathway without statins (by fixing the state of statins to “0”)
2. Providing statins to the pathway as a perturbation (by setting the state of statins to “1”)
3. After the pathway becomes stable, observing a steady state from the pathway

From this scenario, we sampled L attractors for each case of without statins and with statins. Among 2^{32} possible states with the statins status of “0”, L states are randomly selected as initial states. From each initial state, state transition proceeds according to the specification of the Boolean network model until an attractor is encountered. Because we are using a deterministic Boolean network model, every initial state must arrive at an attractor. Attractors sampled in this way correspond to the set of steady states without statins perturbation. From each sampled attractor, a steady state is randomly chosen with a uniform probability and the perturbation of setting the status of statins to “1” is applied to the chosen state. The state transition proceeds again until an attractor is encountered. Attractors sampled in this case correspond to the set of steady states after perturbation by statins. With sampled attractors, we defined $P(s)$ of a state s in an attractor A as follows:

$$P(s) = \frac{1}{|A|} \times \frac{\text{Frequency of } A}{L}$$

**Without statins**			**With statins perturbation**		
**Attractor**	**Size**	**Frequency**	**Attractor**	**Size**	**Frequency**
A_1	1	49,868	A^s_1	1	49,868
A_2	29	49,547	A^s_2	1	50,132
A_3	33	268			
A_4	33	122			
A_5	33	195			

Table S2. The statistics of sampled attractors. Sampled attractors for each case of without statins and with statins perturbation. Size represents the number of states in an attractor. Frequency indicates the number of initial states that arrived to that attractor.

We sampled $L = 10^5$ states from attractors for each case of without statins and with statins. The statistics of sampled states and attractors are listed in Table S2. Based on the statistics of sampled attractors and $P(s)$, 100 states were sampled from the condition without statins as $\boldsymbol{D}_{No\_statins}$ and 100 states were sampled from the condition with statins perturbation as $\boldsymbol{D}_{statins}$.

Supplementary Figure Legends

Supplementary Figure S1

The summarized gene set expression data of refractory cancer patients. There are 339 contextual gene sets for 113 cancer patient samples of 32 tissue types. The expressions

of genes in each contextual gene set were summarized to one of three discrete values – UP (red), DOWN (green), and NOCHANGE (black).

Supplementary Figure S2

The heat maps of cancer-generic region (A) and tissue specific regions (B) - (E) from the refractory cancer contextual gene set interaction network. (A) Cancer-generic. (B) Melanoma. (C) Pancreas. (D) Ovary. (E) Breast. The numbers are IDs of contextual gene sets. The corresponding contextual condition of each contextual gene set is marked with black color above the heat map. Over-expression is colored with red and under-expression is colored with green.

Supplementary Figure S3

The summarized gene set expression data of GBM samples from TCGA. There are 316 contextual gene sets for 202 GBM patient samples of four subtypes. The expressions of genes in each contextual gene set were summarized to one of three discrete values – UP (red), DOWN (green), and NOCHANGE (black).

Supplementary Figure S4

The heat maps of GBM-generic region (A) and GBM subtype-specific regions (B) – (G) from the TCGA-GBM contextual gene set interaction network. (A) GBM-generic. (B) and (C) Classical. (D) Mesenchymal. (E) Neural. (F) and (G) Proneural. The corresponding contextual condition of each contextual gene set is marked with black color above the heat map. Over-expression is colored with red and under-expression is colored with green.
