# Supplementary figures and images for: Learning contextual gene set interaction networks of cancer with condition specificity
Source: BMC Genomics. 2013 Feb 19;14:110. doi: 10.1186/1471-2164-14-110 (PMC3644282; doi:10.1186/1471-2164-14-110)

Contextual gene set

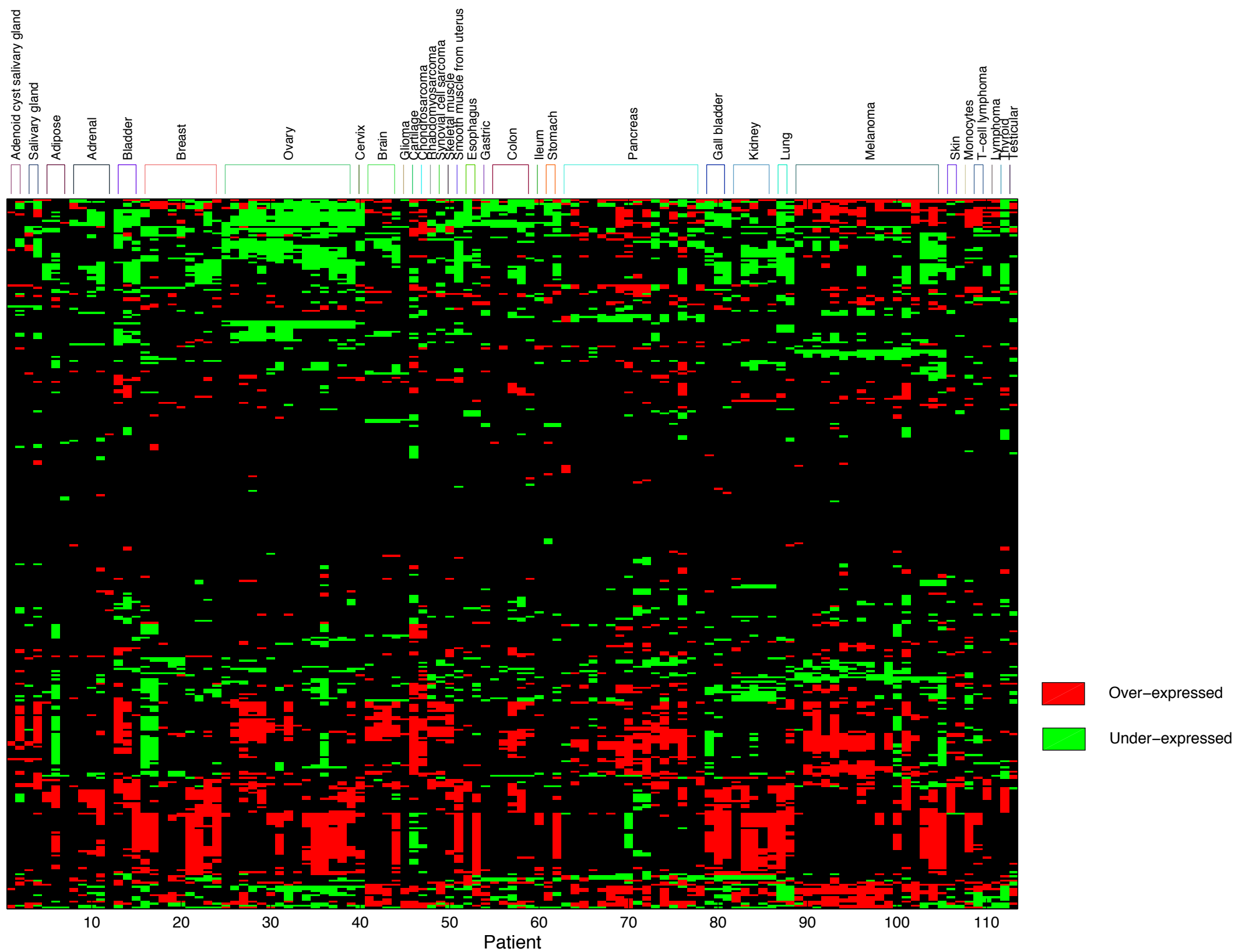

Supplement: Additional file 2 — Figure S1. The summarized gene set expression data of refractory cancer patients. [file 1471-2164-14-110-S2.pdf]

(A)

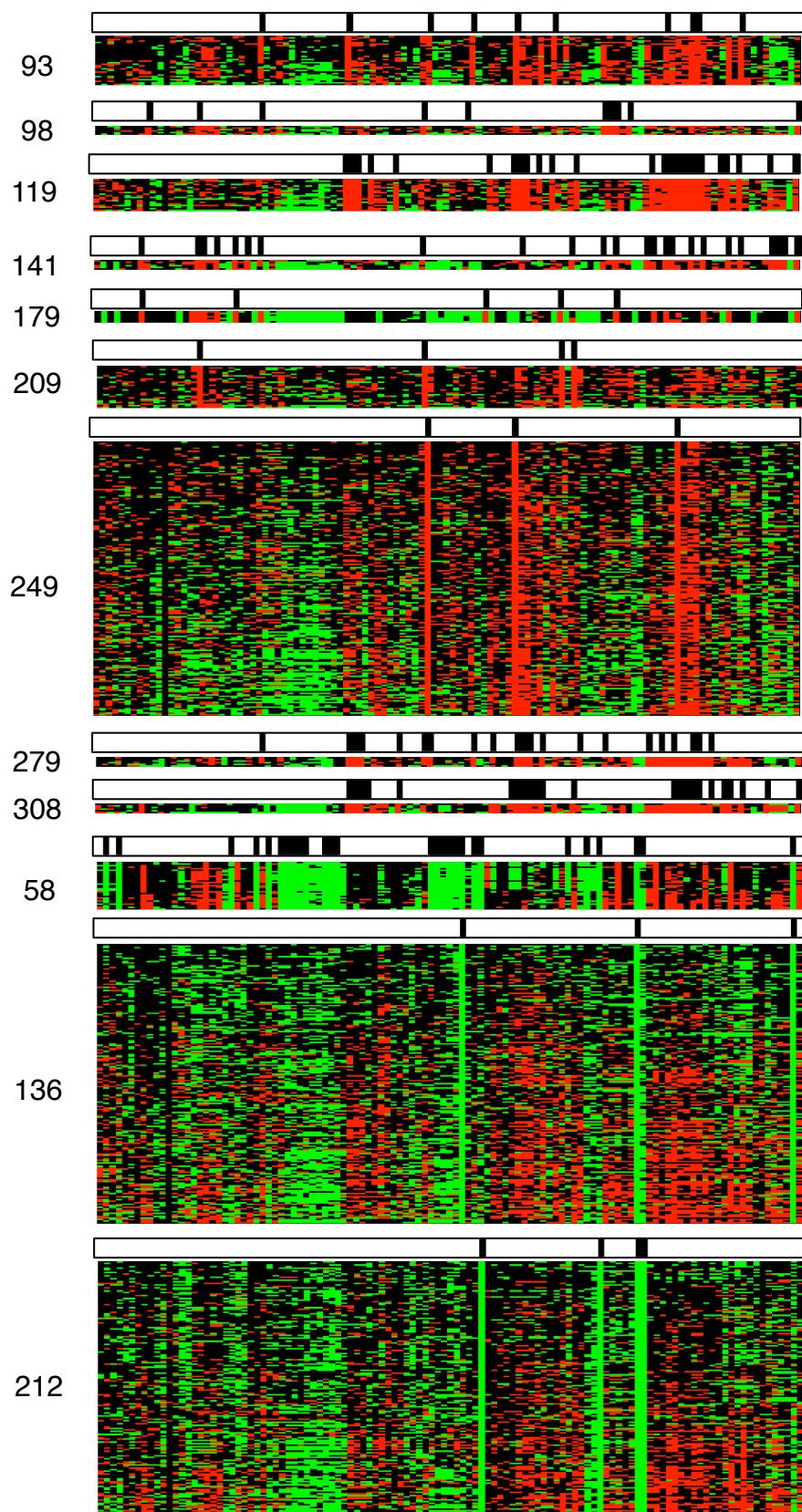

(B)

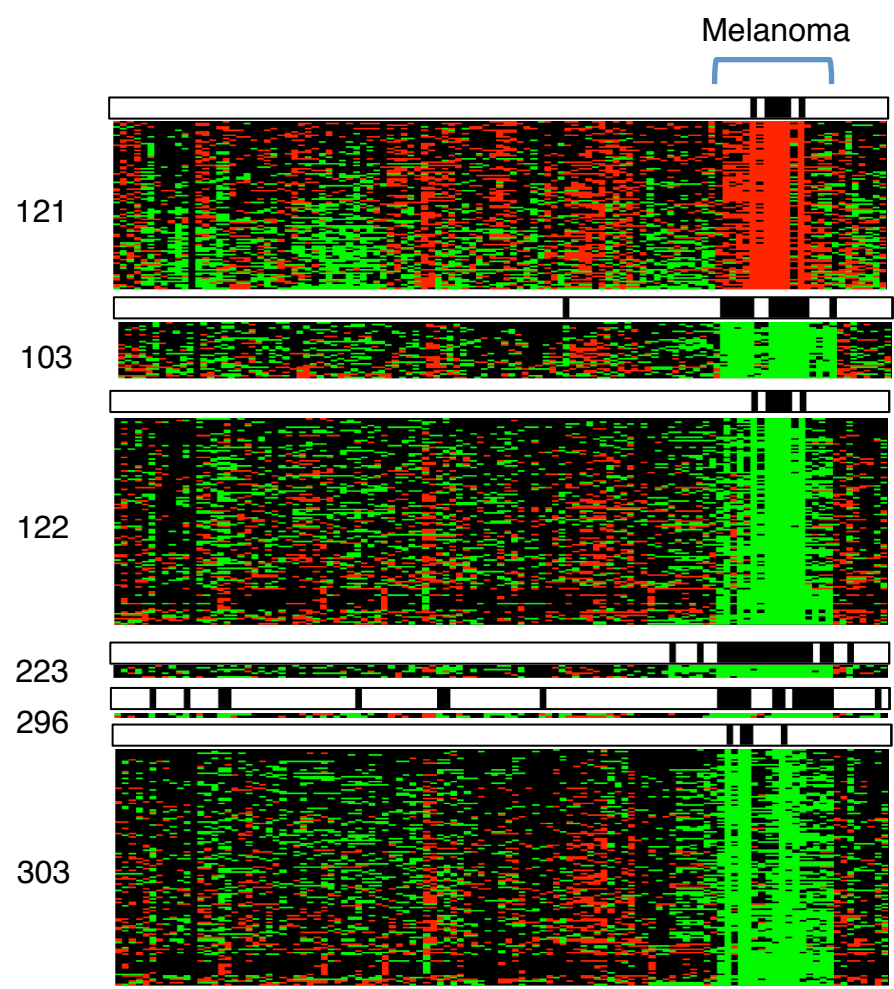

(C)

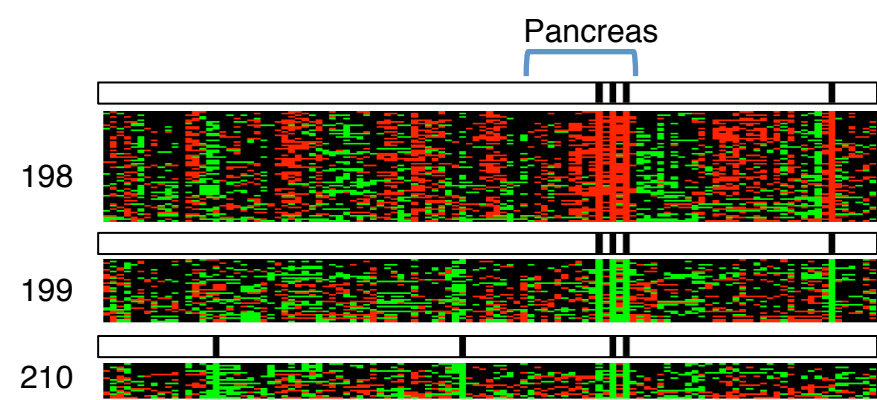

(D)

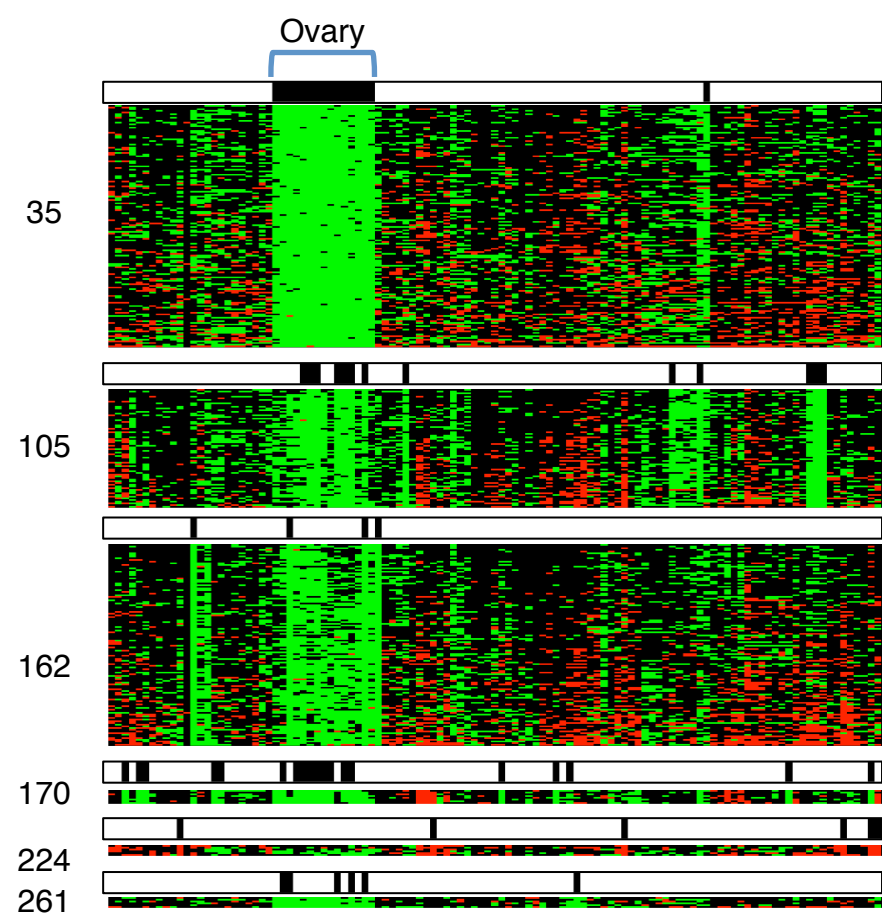

(E)

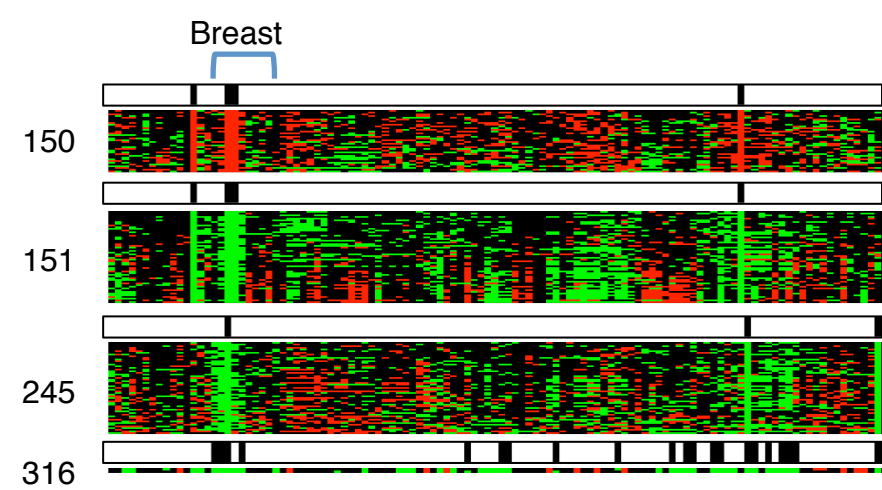

Supplement: Additional file 4 — Figure S2. The heat maps of cancer-generic region and tissue specific regions from the refractory cancer contextual gene set interaction network. [file 1471-2164-14-110-S4.pdf]

**(A)**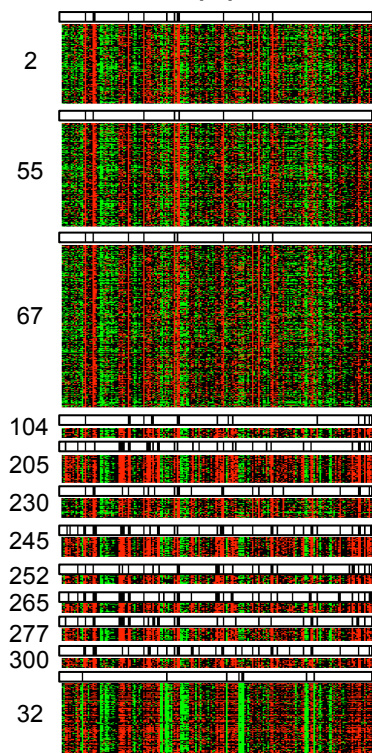**(B)**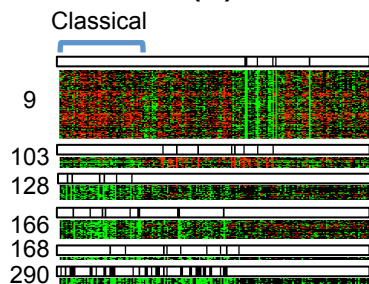**(C)**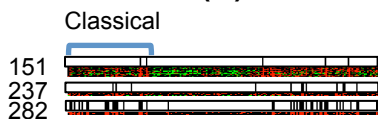**(D)**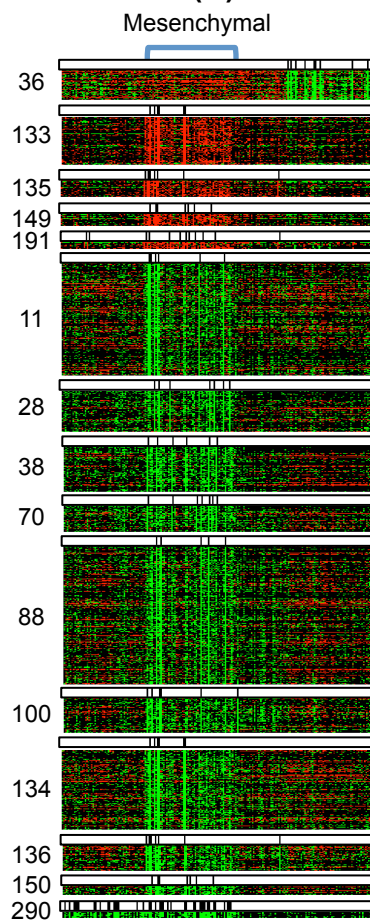**(E)**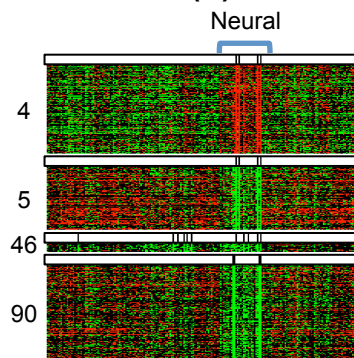**(F)**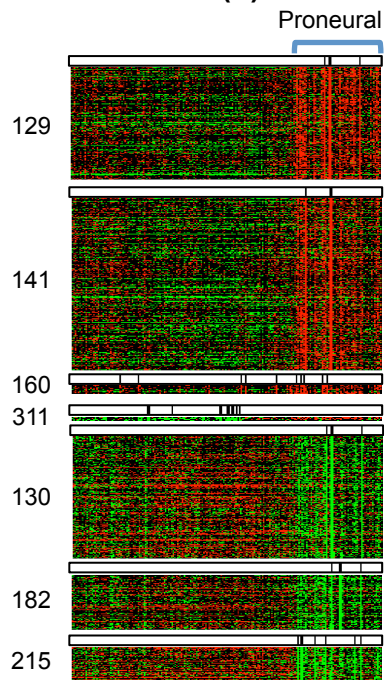**(G)**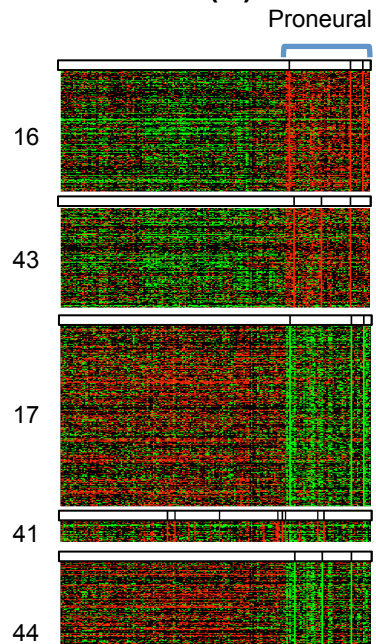

Supplement: Additional file 6 — Figure S4. The heat maps of GBM-generic region and GBM subtype-specific regions from the TCGA-GBM contextual gene set interaction network. [file 1471-2164-14-110-S6.pdf]
