# Supplementary material for: Learning contextual gene set interaction networks of cancer with condition specificity
Source: BMC Genomics. 2013 Feb 19;14:110. doi: 10.1186/1471-2164-14-110 (PMC3644282; doi:10.1186/1471-2164-14-110)
Supplement: Additional file 5 — Figure S3. The summarized gene set expression data of GBM samples from TCGA. [file 1471-2164-14-110-S5.pdf]

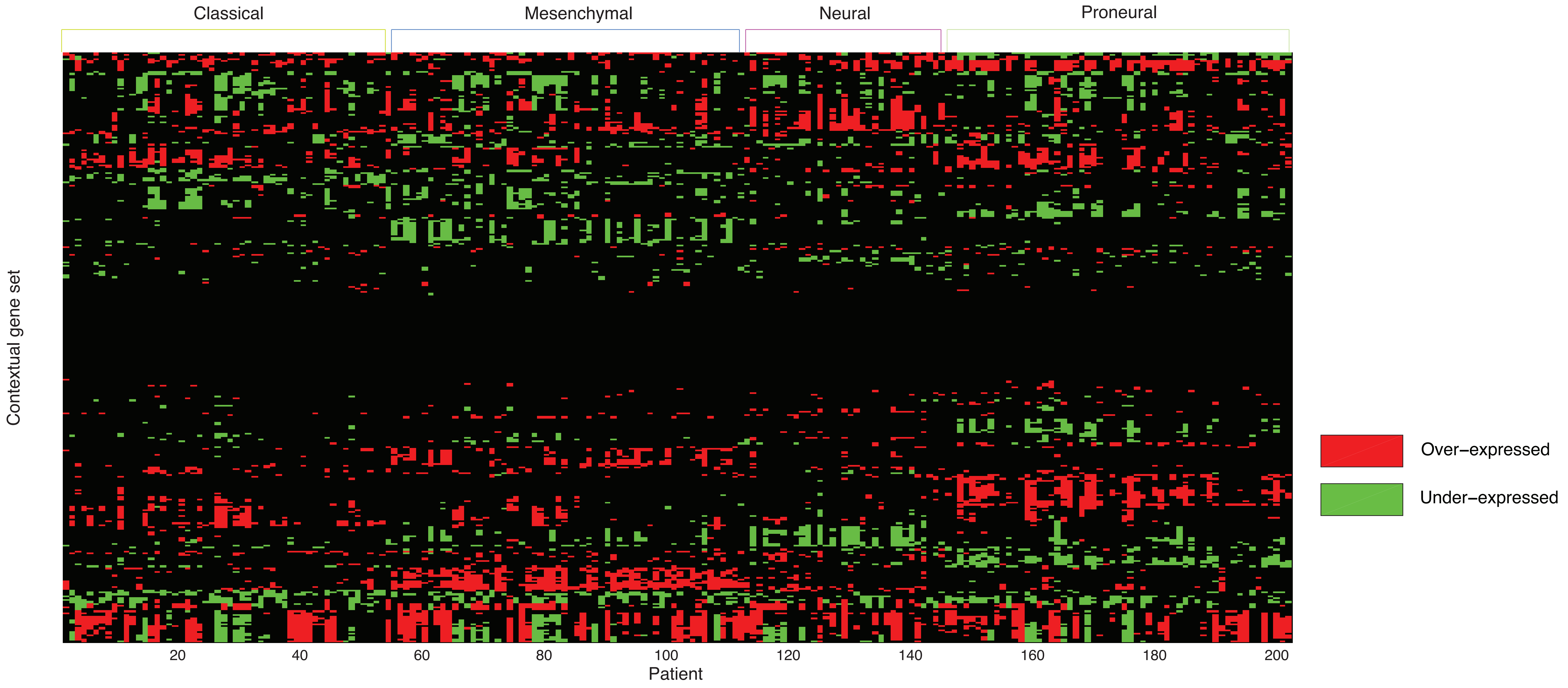
Classical
Mesenchymal
Neural
Proneural
Contextual gene set
20
40
60
80
100
120
140
160
180
200
Patient
Over–expressed
Under–expressed
